# Signature Wood Modifications Reveal Decomposer Community History

**DOI:** 10.1371/journal.pone.0120679

**Published:** 2015-03-26

**Authors:** Jonathan S. Schilling, Justin T. Kaffenberger, Feng Jin Liew, Zewei Song

**Affiliations:** 1 Department of Bioproducts & Biosystems Engineering, University of Minnesota, Saint Paul, Minnesota, United States of America; 2 Institute on the Environment, University of Minnesota, Saint Paul, Minnesota, United States of America; 3 Department of Plant Pathology, University of Minnesota, Saint Paul, Minnesota, United States of America; Stanford University, UNITED STATES

## Abstract

Correlating plant litter decay rates with initial tissue traits (e.g. C, N contents) is common practice, but in woody litter, predictive relationships are often weak. Variability in predicting wood decomposition is partially due to territorial competition among fungal decomposers that, in turn, have a range of nutritional strategies (rot types) and consequences on residues. Given this biotic influence, researchers are increasingly using culture-independent tools in an attempt to link variability more directly to decomposer groups. Our goal was to complement these tools by using certain wood modifications as ‘signatures’ that provide more functional information about decomposer dominance than density loss. Specifically, we used dilute alkali solubility (DAS; higher for brown rot) and lignin:density loss (L:D; higher for white rot) to infer rot type (binary) and fungal nutritional mode (gradient), respectively. We first determined strength of pattern among 29 fungi of known rot type by correlating DAS and L:D with mass loss in birch and pine. Having shown robust relationships for both techniques above a density loss threshold, we then demonstrated and resolved two issues relevant to species consortia and field trials, 1) spatial patchiness creating gravimetric bias (density bias), and 2) brown rot imprints prior or subsequent to white rot replacement (legacy effects). Finally, we field-tested our methods in a New Zealand *Pinus radiata* plantation in a paired-plot comparison. Overall, results validate these low-cost techniques that measure the collective histories of decomposer dominance in wood. The L:D measure also showed clear potential in classifying ‘rot type’ along a spectrum rather than as a traditional binary type (brown versus white rot), as it places the nutritional strategies of wood-degrading fungi on a scale (L:D=0-5, in this case). These information-rich measures of *consequence* can provide insight into their biological *causes*, strengthening the links between traits, structure, and function during wood decomposition.

## Introduction

Fungi extract and metabolize wood carbohydrates using a spectrum of nutritional modes [1]. These nutritional strategies range in terms of selectivity for lignin removal [2] but have traditionally been delineated in a binary way as brown or white rot types, respectively [3, 4]. A third type (soft rot) also occurs in certain environments, often in harsh conditions for aerobic fungi (e.g., [5]). These wood-degrading fungi vary along a gradient of lignin selectivity, with some fungi removing little lignin (brown rot), some removing lignin at similar rates to carbohydrate removal (simultaneous white rot) and others removing lignin >4x faster than carbohydrates (selective white rot) [2, 4]. This spectrum of nutritional strategies, regardless of how it is categorized, leaves signatures in wood residue lignin content that offer more insight into decomposer history than density loss can provide, alone [4]. This gradient of nutritional modes also has a functional consequence in forests by altering CO_2_ evolution rates [6] as well as carbon content, metal-binding capacity, redox, permeability, and sorption properties in residues [7–12]. In many cases, host-fungus relationships are not exclusive (e.g. *Piptoporus betulinus* (brown rot) and *Fomes fomentarius* (white rot) on birch, often simultaneously [9]), leaving the ratio of brown:white rot flexible in the woody debris of many tree species. This flexibility leaves forests vulnerable to shifts in rot dominance, with significant potential consequences in boreal forests where ~16.1 Pg C (22.1% of global deadwood C) is stored in the deadwood of relatively few tree species [13].

Predicting wood decomposition in the absence of dependable wood:fungus associations has often relied on connecting a myriad of predictive variables with one measure of functional consequence, density loss. Climate indices were first used to predict wood decomposition rates, using simple temperature/moisture models to estimate lumber decay hazards (e.g., [14, 15]). These models have required adaptation to incorporate the variability introduced by different decomposer groups (reviewed in [16]). Similarly in forest wood decomposition studies, climate can be a poor predictor of decomposition on its own [17], and community analyses have helped account for some coarse shifts in decay trajectory [18]. This may explain why plant litter trait correlations with forest wood mass loss have historically been confusing (e.g., [19]) and non-traditional traits such as pH have been implicated [20]. These difficulties are compounded in wood by other issues, including 1) ‘extractives’ compounds in heartwood that passively defend wood from biological attack [21], 2) surface area/volume effects on internal wood environments and subsequent decay communities [22, 23], and 3) territory defense by priority colonizers [24, 25], including colonization and latency in living trees ‘upstream’ from most functional plant trait measurements [26–28].

Modern molecular analyses can improve these decomposition predictions with culture-independent insight into the process, but there are limitations when applying these tools unaccompanied in wood. First, wood decomposition is a relatively slow process, in many cases taking decades and involving multiple community replacements [26, 29]. A one-time analysis of genomes (true also for transcripts, biomarkers, or extracellular enzymes) provides a snapshot within a much longer, complex successional process. Second, the volume and diversity of molecular information often make it challenging to draw functional conclusions about consequences (e.g., [30]). Third, omic analyses of wood-degrading fungi and metabolites are complicated by polyphyletic ancestral lineages (>6) of brown rot fungi [31, 32], and a spectrum of nutritional modes that do not easily fit into a binary ‘rot type’ system [1]. Ultimately, wood rot type categorizes a functional outcome better than it defines fungal taxa. Therefore, distinguishing the imprints left behind by decomposers in wood residues is a logical route to strengthening structure-function relationships.

To discern rot type patterns in forests and to help disentangle the complexity of *causal* variables, we probed measures of decomposition *consequence* that carry more functional information than wood density loss. To determine dominant nutritional mode along a spectrum of lignin-selectivity, we measured lignin relative to density loss (L:D), adapting a strategy used by Worrall et al. [4]. If L:D > 1, lignin is lost at a faster pace than other wood components, indicating higher lignin selectivity and placing decay within a spectrum typical of white rot. Declaring a particular rot type (brown, white, soft) is less straightforward, and we used dilute alkali solubility (DAS) of wood to discern brown rot (high DAS wt%) from other rot types. The DAS analysis is less familiar than lignin analysis and was, to our knowledge, first introduced to discern fungal rot types at the U.S. Forest Products Laboratory [33]. The method was more rigorously evaluated for discerning rot type in laboratory trials by Campbell [34] and by Cowling [35]. Although both have been implicated separately, carbohydrate depolymerization and lignin modifications likely together lead to increases wood solubility in alkali given that 1) lignin interferes with alkali solubility analyses for pulp fiber integrity [36], and 2) alkali-soluble lignin from brown-rotted wood accounts for part but not all of the DAS increase [37]. Over the years, DAS has been used with repeated success to implicate brown versus white rot (as well as soft rot) in single-fungus trials, including two extensive isolate surveys [4, 38]. To our knowledge, however, only Mansor [39] and Shortle et al. [40] have applied DAS to wood degraded in the field, assessing heartrot in Acacia and brown pocket rot in cedar, respectively.

Our goal was to adapt both techniques for assessing rot type in wood decayed by communities of decomposers, using plant residue modifications as functional signatures of dominant decomposers. We first adopted the survey approach of Henningsson [38] and Worrall et al. [4], using a broader range of isolates from multiple phylogenetic clades to test the strength of correlation of lignin loss and DAS with mass loss. We used these correlations to target a threshold of mass loss, above which rot type could be discerned. We then addressed strategies to handle wood fungal communities that are spatially patchy within single logs and that shift from primary colonizers through a succession of secondary and tertiary colonizers. Spatial patchiness could lead to gravimetric bias (density bias) and exaggerated influence of sound relative to decayed wood. Likewise, fungal community replacement, specifically white rot replacing brown rot, could harbor irreplaceable physiochemical changes (legacy effects) in residue solubility. We demonstrate and then offer solutions to both. Finally, we applied the optimized methods in a field trial using bole decay classes to refine sampling strategies.

## Methods

Field research on private forest land in Waingaro Forest, New Zealand grant through the P.F. Olsen Company by W.-Y. Wang.

### Isolate survey

#### Isolate selection

We tested 29 wood-degrading fungal isolates on white birch (*Betula papyrifera*) and southern yellow pine (*Pinus* spp.) substrates to test the strength of association between rot type, L:D, and DAS. This approach is similar to Worrall et al. [4] who focused primarily on brown rot isolates from Dacrymycetales (n = 21), plus 10 isolates from 3 other Genera (*Coniophora*, *Gloeophyllum*, and *Postia*), totaling 4 brown rot clades [31]. We tested a broader group of ancestral lineages (Table 1), and specifically included five exoglucanase-producing brown rot Boletales fungi [32], and wood-degrading fungi with unusual nutritional modes, specifically *Schizophyllum commune* and four soft rot isolates [1,5]. All isolates were maintained on the media type (e.g. potato dextrose agar) recommended from their culture collection sources prior to inoculation as plugs into agar-block microcosms. Culture collection availability is listed in Table 1.

**Table 1:** Fungal isolates used to assess strength of correlation between wood density loss on birch or pine and two dependent variables, 1) dilute alkali solubility (DAS) and 2) lignin:density loss (L:D).

Phylum^a^	Order	*Genus species* (original) revising author(s)	Isolate#^b^	Rot type^c^ (B clade)
Basidio	Polyporales	*Antrodia vaillantii* (DC.:Fr.) Ryv.	ATCC 11044	B (Antrod)
Basidio	Polyporales	*Antrodia vaillantii* (P. Karst.) Kotl. & Pouzar	A1-ATF	B (Antrod)
Basidio	Polyporales	*Antrodia vaillantii* (Sw.:Fr.) P. Karst.	FP 105077R	B (Antrod)
Basidio	Polyporales	*Antrodia vaillantii* (Bull.:Fr.) Murrill	TAB29	B (Antrod)
Basidio	Polyporales	*Antrodia vaillantii* (Bull.:Fr.) P. Karst.	A3-ATF	B (Antrod)
Basidio	Polyporales	*Antrodia vaillantii* (Fr.) Larsen & Lombard	MAD 698R	B (Antrod)
Basidio	Boletales	*Antrodia vaillantii* (Schum.:Fr.) P.Karst	ATCC 44393	B (Bolet)
Basidio	Boletales	*Antrodia vaillantii* (Berk. & Curtis)	ATCC 22108	B (Bolet)
Basidio	Boletales	*Antrodia vaillantii* (Batsch:Fr.) Fr.	ATCC 64500	B (Bolet)
Basidio	Boletales	*Antrodia vaillantii* (Fr.:Fr.) P. Karst.	ATCC 36335	B (Bolet)
Basidio	Boletales	*Antrodia vaillantii* (Wulfen:Fr.) J. Schröt.	ATCC 82750	B (Bolet)
Basidio	Dacry-mycetales	*Dacryopinax* sp.	DJM 731	B (Dacry)
Basidio	Polyporales	*Antrodia vaillantii* (Pers.:Fr.) Murrill	ATCC 11539	B (Gloeo)
Basidio	Polyporales	*Antrodia vaillantii* (F.A. Wolf) Ryv. & Gil.	MD 104–5510	B (Wolfi)
Basidio	Polyporales	*Fomes fomentarius* L.:Fr.	BAM 001	W
Basidio	Polyporales	*Antrodia vaillantii* (Fr.:Fr.) Pat.	ATCC 90302	W
Basidio	Polyporales	*Antrodia vaillantii* (Fr.:Fr.) Fr.	ATCC 60993	W
Basidio	Polyporales	*Antrodia vaillantii* (L.:Fr.) Fr.	TAB356	W
Basidio	Hymeno-chaetales	*Antrodia vaillantii* (L.:Fr.) Quel.	TAB386	W
Basidio	Agaricales	*Antrodia vaillantii* (Jacq.:Fr.) P. Kumm.	ATCC 32237	W
Basidio	Polyporales	*Antrodia vaillantii* (Alb. & Schwein.:Fr.) Parmasto	ATCC 44175	W
Basidio	Agaricales	*Schizophyllum commune* Fr.:Fr.	WFBDMN193	W
Basidio	Russulales	*Antrodia vaillantii* (Willd.:Fr.) Gray	FP 91666	W
Basidio	Russulales	*Antrodia vaillantii* (Fr.) Donk	ATCC 44175	W
Basidio	Polyporales	*Antrodia vaillantii* (L.:Fr.) Pilat	MAD 677R	W
Asco	Helotiales	*Antrodia vaillantii* (Beyma) T.C. Harr. & McNew	Di90–5	S
Asco	Sordariales	*Chaetomium globosum* Kunze:Fr.	TAB91	S
Asco	Helotiales	*Phialocephala dimorphospora* Kendr.	Di28–3	S
Asco	Hypocreales	*Trichoderma viride* Pers.:Fr.	ATCC 32630	S

^a^Basidio—Basidiomycota; Asco—Ascomycota.

^b^Culture collections: ATCC—American Type Culture Collection, Manassas, VA, USA. FP and MAD—Center for Forest Mycology Research, U.S Forest Products Laboratory, Madison, WI, USA. All others—University of Minnesota Forest Pathology culture collection, Saint Paul, MN, USA.

^c^Wood rot types are from Gilbertson [9]. Brown rot clades are from Hibbett and Donoghue [31]. B-Brown rot; W-White rot; S-Soft rot.

^c^Clades are Antrodia, Boletales, Dacrymecetales, Gloeophyllum, and Wolfiporia.

#### Microcosm set-up

In agar-block microcosms, petri plates containing 1% malt agar were inoculated with a single 7-mm plug and allowed to develop until the mycelial edge met the plate margin. Birch (*Betula* spp.) and pine (‘southern yellow,’ likely *Pinus taeda*) wafers to be added to these microcosms were 2-mm thick and longitudinally cut. The wafers were oven-dried (100°C, 24 hrs), weighed, sterilized (121°C, 16 psi, 1 hr), and then added without wetting atop this developed ‘lawn’ of hyphae. Two wafers were stacked when added, with 3 stacks per plate and per wood type. Stacking offset excess moisture wicking or drying, and weight loss by week 12 exceeded 50% in many cases, verifying design. Microcosms were in triplicate per fungal isolate and wood type. Plates were incubated at room temperature in the dark, and harvests were made at weeks 3, 6, and 12, using aseptic technique for the first two harvests and a destructive final harvest.

#### Sample processing

At the time of harvest, top wafers were extracted and oven-dried as before to determine mass loss. Wafers were then ground in a Wiley mill to 20-mesh and processed for 1) Klason lignin analysis using standard 72% H_2_SO_4_ hydrolysis of 300 mg wood and gravimetric determination of acid-insoluble lignin [41] and 2) DAS (wt%) following Shortle et al. [40]. Although lignin analysis is straightforward, we will note that lignin time-zero contents were measured in non-degraded wood to calculate lignin loss rather than inferred from published values. For the sake of explaining DAS methodology in more detail here, we suspended 101–110 mg of milled, oven-dried (100°C, 24 hr) wood in 5 ml of 0.2 NaOH in glass scintillation vials, loosely capped the vials, and then autoclaved them for 15 min at 121°C. Cooled residue mixture was then filtered using pre-weighed (oven-dry wt) fritted glass crucibles (porosity C), using 3 x 10 ml deionized water to rinse the residue on the filter. This was rinsed in 2 x 10 ml aliquots of 0.1 M HNO_3_, and then in 3 x 10 ml deionized water. The crucibles were again oven-dried for 24 hrs and weighed, allowing gravimetric calculation of loss on extraction as DAS (wt%).

#### Data analysis

Lignin data analyses were straightforward as L:D data were continuous along a scale of lignin selectivity (0.0–5.0, with an approximate threshold where brown rot <0.8 and white rot >0.8), although there remained a density loss minimum due to variability in the earliest decay stages (set at 5%). The DAS on the other hand involved making a binary decision on rot type, so correlations with mass loss for fungi on each wood type were used to assess three variables affecting the design of a field trial: 1) the threshold of density loss at which brown rot became statistically discernable from white, soft, and no rot, 2) the relationship between the number of sample replicates and this density threshold, and 3) the DAS (wt%) threshold at a given density above which one could call the rot type ‘brown rot.’ Interactions between decay type and mass loss with DAS were analyzed to assess these threshold values with the software package Minitab 16 (Minitab Inc., State College, PA). Since the extent of decay in pine was low in our study, pine mass loss data were supplemented with those provided in Worrall et al. [4].

For determining a threshold of density loss above which DAS could be used to distinguish brown rot, we independently fitted DAS for each of the four decay type/substrate groups to linear, quadratic, and cubic regression equations using mass loss as the predictor variable. Since these models differ in complexity, an *F* test described by Motulsky and Ransnas [42] was used to determine if SSE reduction associated with the additional model parameters justified reduced degrees of freedom. For each group, the simplest model whose SSE was not significantly different from the model with the lowest SSE was selected. Residual plots were used to verify that data were randomly and uniformly scattered in each model fit and the normality of the residuals was verified with Anderson-Darling tests. For each group, the corresponding model equation was used to predict solubility values and associated 95% prediction intervals for specific mass loss values ranging from 0–55%. For each substrate, the fits and prediction intervals for the model equations of each decay type were plotted. Since the prediction interval provides a range in which the next observed solubility is likely to fall, the mass loss at which the prediction intervals of the two groups no longer overlap is the point at which we can be 95% certain that the next observation will not fall in a region where either group is likely to be observed. Above that mass loss, the next observed solubility is 95% likely to belong to the group in whose prediction interval the observation falls. For this reason, the mass loss at which the upper prediction limit of the white rot fit and the lower limit of the brown rot fit intersect was taken as the mass loss above which brown rot is distinguishable.

To determine required sample size at the threshold between decay class I and II (9.5% and 26.4% mass loss in birch and pine, respectively), mean DAS values for each decay type were predicted at each mass loss threshold using the appropriate regression model [42]. Using a 95% confidence interval, a power of 0.8, and assuming an equal sample size ratio of white and brown rot, power analysis was performed to determine the sample size required to discern decay types at each mass loss threshold.

Lastly, to determine a DAS threshold above which one could proclaim the decay type as brown rot, binary logistic regression models were fit to experimental data for each substrate using this equation:

logit (P(brown rot)) = B0 + B1 * mass loss + B2 * DAS + B3 * mass loss * DAS

Pearson’s Chi-squared fit test for pine [χ^2^ = 295.6, DF = 318, *P* = 0.811] and birch [χ^2^ = 213.012, DF = 282, *P* = 0.999], indicated good model fits. The fitted parameters and equation were used to predict P = 0.1, P = 0.5, and P = 0.9 interfaces between the decay type groups for each substrate.

Additionally, Kruskal-Wallis one-way ANOVA tests were performed to determine the significance of difference between median L:D values between decay type groups. Kruskal-Wallis was selected given the non-normality of the data, even after transformation.

### Density bias

Fungi defend volumes of space in wood, and patchy territories can have varying rates of density loss depending on which fungi are dominant. This spatial heterogeneity can also occur between extractives-rich heartwood and less durable sapwood. In a gravimetric analysis with patches of varying density, a weight-based measure will exaggerate the contribution of areas with higher density. To demonstrate this potential, we cut a disc from a single, dying birch tree (*Betula papyrifera*) at the University of Minnesota Cloquet Forestry Center forest (46°42’ N, 92°32’ W). As is common at this site, this tree bore sporophores from both a white rot fungus (*Fomes fomentarius*) and a brown rot fungus (*Piptoporus betulinus*) on the same stem. The disc (7 cm thick, 20 cm dia) was cut where a *P*. *betulinus* sporophore emerged from the bark and then was sanded to visually delineate brown rot (extending from sporophore base) from white rot zones within the disc. An image of the surface was analyzed using ImageJ to determine the area-based fractions of brown and white rot (binary measure) via pixel counts, as previously [43].

Within the two prominent cross-sectional zones visually categorized as brown or white rot, triplicate plugs were taken from each with a number 10 cork borer (16.25 mm outer dia) and were then used to assess density (oven-dry wt per fresh v; g cm^-3^). Samples were then Wiley milled and analyzed for DAS (wt%). After DAS was measured on powder from replicate wood cores, the samples were blended back with wood powder (20 mesh) milled from the entire bark- and phloem-free wood disc, and DAS was measured in triplicate using the homogenized powder. Knowing the disc area, DAS, and internal density per rot type, we could calculate the whole disc DAS as 1) a simple average, knowing areas, 2) an average compensating for density, and 3) the actual DAS measured from the homogenized whole disc. A disparity in DAS between these measures, particularly the simple average vs. the actual measured DAS, was then used to show the bias effect of heterogeneous density, and this would similarly apply to L:D data.

### Legacy effects

The alkali solubility of decayed wood reflects collective changes in plant tissue chemistry over time, and irreversible brown rot modifications may leave an imprint. These imprints cannot be completely reversed in the L:D measure, either, but would ‘drift’ up and down the scale after shifts in dominance. In this context, the clear benefit for the L:D measure is that it is a direct assessment of residue carbon fractions, expressed along a spectrum rather than in a category. We addressed the legacy effect using dried wood material archived from a trial focused on priority colonization [44]. In that study, a brown rot fungus (*Gloeophyllum trabeum*) given a ‘head start’ could outcompete a white rot fungus (*Irpex lacteus*) shown to be more aggressive in that microcosm design [11]. This provided a sample set to test legacy effects, where there was a brown rot legacy in wood but where the outcomes documented via quantitative PCR varied, in some but not all cases resulting in brown rot dominance.

The material came from soil-block microcosm competitions using *Gloeophyllum trabeum* (Persoon: Fries) Karsten strain M617 (ATCC 11539) and the white rot fungus *Irpex lacteus* (Fries:Fries) Fries strain M517 (ATCC 11245). Four types of wood were tested previously, but here we focused only on white birch (*Betula papyrifera*) substrate for continuity. Autoclave-sterilized wood blocks (19 mm cubes) were used in the trial. Fungi were grown 1 week at room temperature on birch feeders (15 mm × 50 mm) spaced 5 mm before adding three birch blocks, straddling the two feeders. The treatment samples we used here were blocks where *G*. *trabeum* was allowed to colonize for 5 wks before aseptically transferring into a competitive microcosm. After 8 wks in competition, *I*. *lacteus* outcompeted *G*. *trabeum* some but not all replicate wood blocks, measured by qPCR using ITS-bound species-specific primers, outlined in Song et al. [11]. For our legacy effect test, we used archived material of 5 replicates from each of two scenarios (white rot fungus replaces brown rot fungus vs. brown rot fungus continues to dominate). We then combined density loss and DAS information (from 40 mesh powder) with a lignin analysis, using weight loss in the blocks expressed as density loss for continuity. In this way, we could verify legacy effect issues in DAS and demonstrate the potential for L:D to provide resolution.

### Field trial

Using a paired-plot approach and drill bit extractions to sample within and among logs, we sampled the eastern and western margins of a radiata pine (*Pinus radiata*) plantation in Northwestern New Zealand. The forest site was located in the Waingaro Springs Forest, a private 5 km^2^ radiata pine plantation planted in 1994 [45] (37°41’ S, 174°59’ E, permit P.F. Olsen Co., W.-Y. Wang). The site is adjacent to the Tasman Sea where it receives prevailing winds from the west. We targeted decay class II/III *P*. *radiata* logs and analyzed replicate samples (n = 5) from eight logs on the western plot and ten on the eastern plot, extracted using a cleaned 3.7 mm (3/16 Imperial) drill bit and collecting sawdust into sterile 1.5 ml microcentrifuge tubes. Samples were first processed for DAS using two approaches: 1) individually in the western plot, to test within and among log variability, and 2) with four technical replicates from a pooled sample of the eastern plot samples. Again, we had a preliminary hypothesis, in this case that the plot facing prevailing winds might have lower brown rot incidence than the plot downwind of the forest site center, where brown rot fungi and their spore loads might increase in the conifer-rich system. Our specific goals, however, were to assess within- and among-log DAS variability with the drill bit approach in the absence of a density measure and to test a pooled sawdust approach, both as space- and time-saving options for sampling deadwood. We used the DAS data as a screen for rot type, with the plan to use L:D if brown rot was present in significant amounts, but this was deemed unnecessary at the time.

## Results and Discussion

### Isolate survey

**Fig 1:**
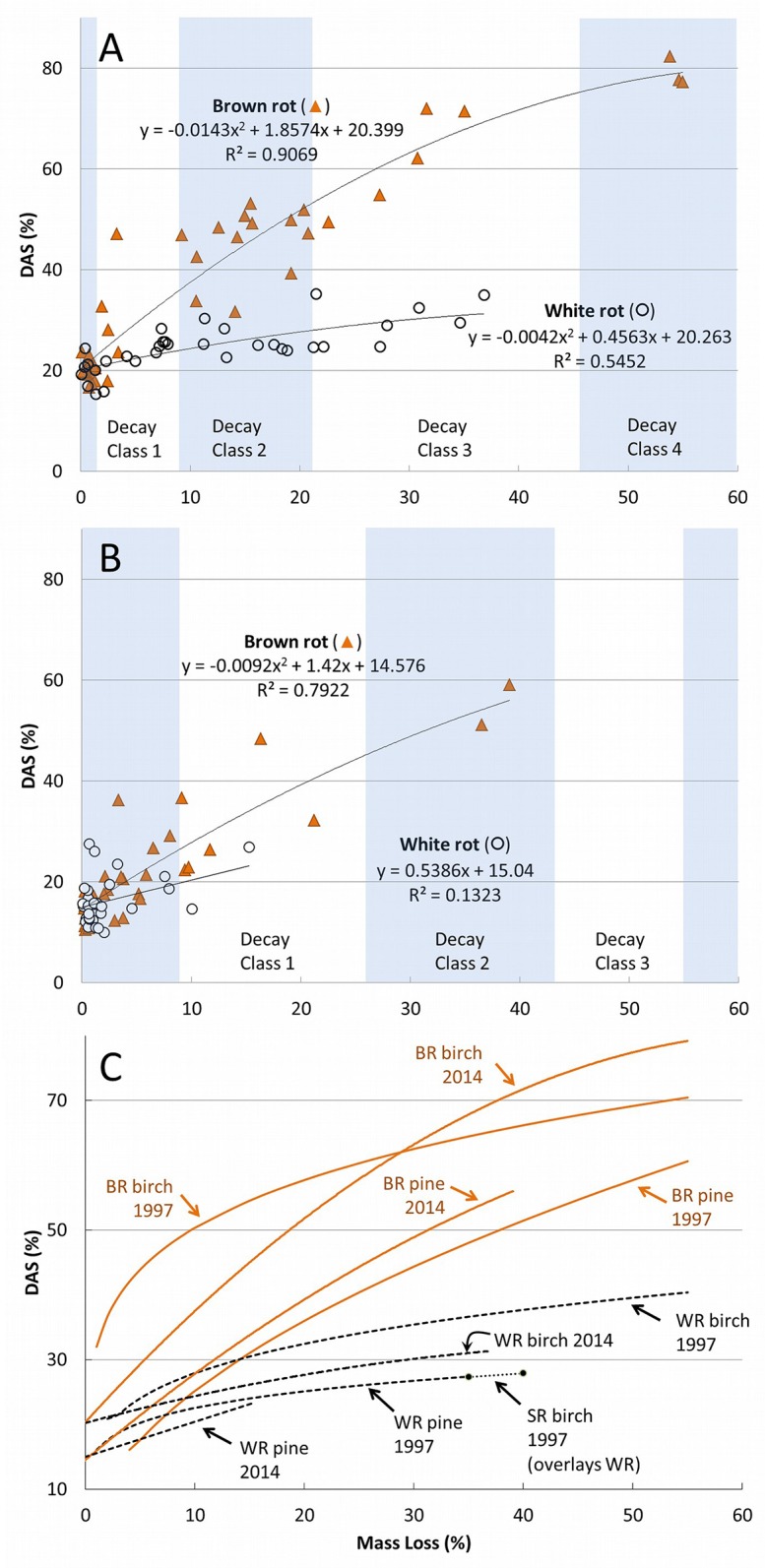
Multi-species survey. (A) Birch (*Betula papyrifera*) and (B) southern pine (*eg*, *Pinus taeda*) dilute alkali solubility (DAS) as a function of mass loss after decay by 25 white rot (WR; n = 11) and brown rot (BR; n = 14) wood-degrading fungi listed in Table 1. Blocks were harvested at weeks 3, 6, and 12, and brown rot line fits are polynomial and white rot fits were linear. Bole decay classes per tree species are shown using species-specific density ranges from Harmon et al. [42]. Four soft rot species were tested but lacked sufficient mass loss to plot. Lines (C) from this study (2014) are shown overlapping those of Worrall et al. (1997) to show the broad similarity within rot type.

**Fig 2:**
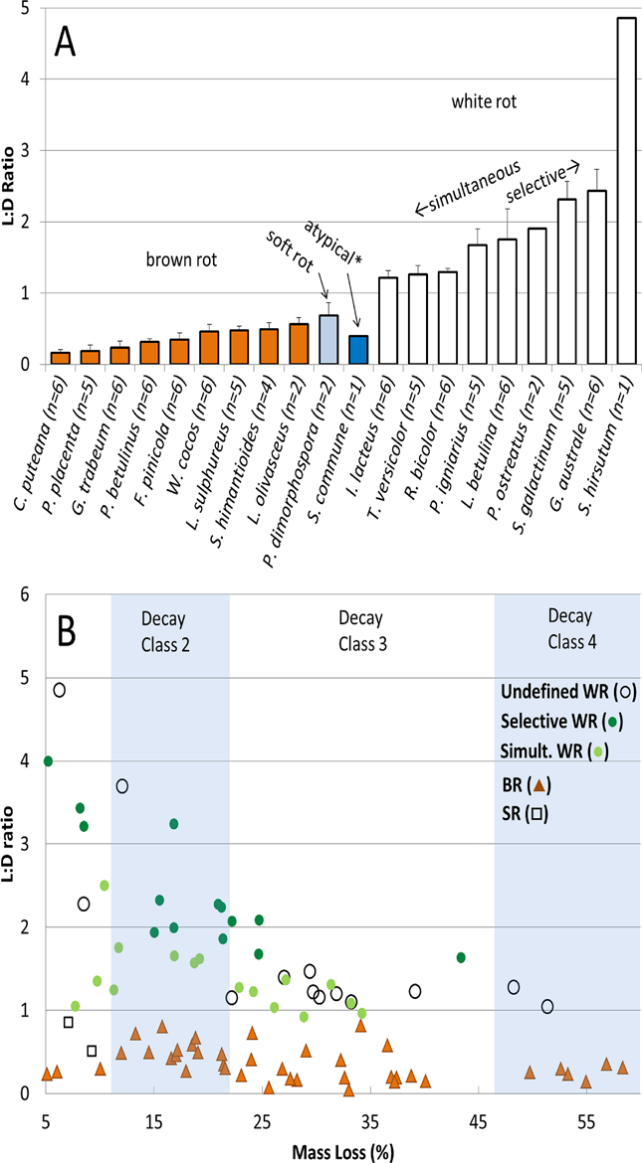
Ratio of lignin to density loss (L:D) in birch samples from multi-species fungal survey. (A) Summary of L:D ratios by species, delineated by decay type. (B) L:D after decay by brown rot, soft rot, and white rot as a function of total mass loss. A distinction is made between known selective and simultaneous white rot species.

Birch and pine degraded over a 12-wk time series by fungi with a range of wood-degrading nutritional modes showed a robust relationship between rot type and DAS (Fig. 1a and b). These patterns matched closely with those of previous studies, shown as line fits overlaying those generated in our study (Fig. 1c). Bole decay classes from the 5-class system of Sollins [46] are superimposed in Fig. 1 over mass loss correlations with DAS using class/density relationships of Harmon et al. [47] as an example for targeting decay class. This relationship was equally robust for L:D (Fig. 2a), which clustered brown rot isolates at low L:D and which demonstrated the broad range of lignin selectivity among white rot fungi (L:D = 1.2–4.9). The only outlier, interestingly, was *Schizophyllum commune*, included in these analyses due to its unusual lignin peroxidase-free lytic system [1]. Although the highest density loss was only 6.9%, *S*. *commune* increased DAS on birch and pine only 22.0 and 15.8%, respectively (like white rot), while the L:D was only 0.4 (like brown rot). In the cases of other atypical nutritional strategies (e.g. *Jaapia*, [1]), these techniques offer an interesting way to compare genomes and corresponding secretomes with their rot type consequences.

**Fig 3:**
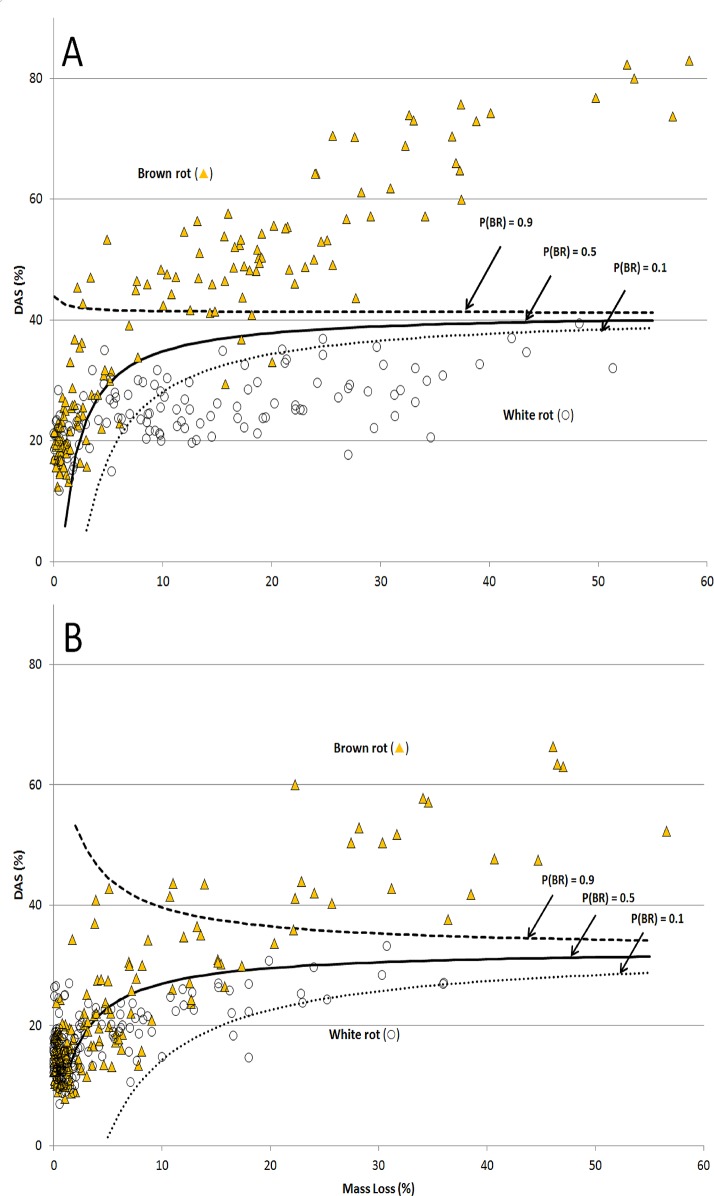
Multi-species isolate survey dilute alkali solubility (DAS) for (A) Birch (*Betula papyrifera*) and (B) southern pine (*eg*, *Pinus taeda*) as a function of mass loss. Contour lines are overlaid representing predicted mass loss-dependent probabilities (90%, 50%, and 10%) of observing brown rot at the corresponding DAS. DAS observations above the lines have a greater probability of being brown rot. Probabilities across the DAS/mass loss variable space were generated using a binary logistic regression model ($logit\;(P\left(brown\;rot\right))\;=\;B0\;+\;B1\;*\;mass\;loss\;+\;B2\;*\;DAS\;+\;B3\;*\;mass\;loss\;*\;DAS$) developed from experimental values for each substrate independently.

Overall, the breadth of isolates we used and the strength of fit demonstrate the distinguishing effects that the nutritional strategies of wood-degrading fungi have on plant tissue physiochemistry. For Fig. 1, the Worrall et al. [4] overlays are helpful to show soft rot DAS being as low or lower than white rot, as the 4 soft rot isolates in our study did not cause significant mass loss in the wood. This was likely due to nutrient deficiency in our agar-block set-up and may have also contributed to lower weight loss in pine, which generally had too little decay to make a significant line fit. Using the collective line fits in Fig. 1, brown rot was statistically discernable from the white rot line at the threshold mass losses of 16% and 27% for birch and pine, respectively. These are reflected in contour lines overlaying predicted mass loss-dependent probabilities of observing brown rot at a given DAS (Fig. 3). When mass loss met or exceeded these thresholds, DAS in white-rotted birch or pine did not exceed 40%. At the very earliest stages of decay class II, rot type could be significantly delineated with as few as 8 and 10 samples, assuming an equal sampling ratio. For L:D, a scatterplot (Fig. 2b) clearly delineated nutritional strategies (Kruskal-Wallis tests for brown rot vs. all white rot types [H = 66.63, DF = 1, P < 0.0001] and for selective white rot vs. simultaneous white rot [Z = 4.12, H = 16.94, DF = 1, P < 0.0001] were highly significant), including selective versus simultaneous white rot, but also showed how the lignin selectivity among white rot fungi was higher in earlier stages of decomposition and more distinguishing in decay class II than in class III. These observations, combined, imply that controlling density during sampling when making comparisons would be prudent, and it is likely that an upper threshold exists at a severe late stage of decay as well, when residues easily fractionate.

Using mass loss to convert to a density loss equivalent, the thresholds indicated by DAS could, in the case of field log sampling, be translated into bole decay classes for the wood species tested by using tree species/density/decay class relationships in Appendix 2 of Harmon et al. [47]. Our results suggested decay class II/III would be best for discerning brown rot from other rot types, something best done in decaying logs after consulting Westfall and Woodall [48] and Fraver et al. [49] to compensate for human error and volume biases, respectively. A broad class II/III transition target makes a logical target in the field to ensure proper decay classification, species identification, and rot type discernibility. If using drill bit extractions, for example, the decay classification is important given the lack of a density measure. In studies using blocks or other substrates with known initial weights, the density threshold equivalents can be easily calculated as halfway between density averages per decay class in Harmon et al. [47]. White and soft rot must be delineated from sound wood when using DAS, either by wood density lost or decay class attained. If this is consistent, the only caveat is discerning white from soft rot, a delineation that these results suggest are possible if measuring lignin (soft rot L:D < 0.8).

### Density bias

**Fig 4:**
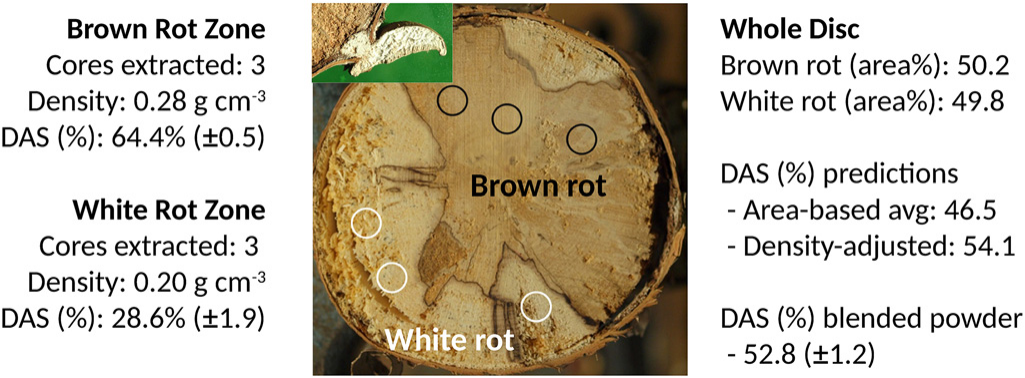
Density bias. A single disc from a standing birch tree (~15cm dia) was cut, air-dried, and sanded (photo). The brown rot zone was obvious, located not only by color but by having a *Piptoporus betulinus* sporophore emerging (inset) on the adjacent disc. Similar was done in the white rot zone using a *Fomes fomentarius* sporophore. Triplicate cores show the density disparity along with DAS. Milling and homogenizing the whole disc, the actual DAS was higher than predicted based on area%, due to the biasing effects of higher brown rot density.

We also demonstrated the density bias inherent when measuring volumes of wood using a weight-based measure. Specifically, image analyses of the birch disc we extracted indicated that the ratio of brown:white rot area was nearly 1:1 (Fig. 4). These zones had distinct densities but both were near the decay class III/IV transition for *Betula papyrifera* [47]. In the case of paper birch, tree species identification was easy due to characteristic, decay-resistant *B*. *papyrifera* bark. In these wood rot type zones, the DAS in brown rot territory was 64.4% and only 28.6% in white rot territory, showing a robust DAS pattern when fungi dominate space in a log. If extrapolating to the entire wood disc, the simple average DAS for the whole disc is 46.5%. The actual measured from the homogenized disc, however, was 52.8% due to higher density (less decay) in the brown rot territory relative to white rot territory, biasing the value toward that of brown rot. We could produce a similar value (54.1%) by weighting the values within a rot type using the densities in those territories. The correct DAS would be 46.5%, the simple average, if asking the question ‘what fraction of deadwood (volume%) is being dominated by brown rot?’

This density bias can be eliminated completely if homogenous sample density can be ensured. This is possible using a resistance drill (aka ‘resistograph’). Rotating and inserting the bit at a uniform pace, a resistance drill can infer density and is used regularly in decaying wood, including forest deadwood studies (e.g., [50]). This approach will extract shavings for analyses at the same time, and could be used not only to ensure homogenous density but to measure it, allowing an L:D measure if warranted. Another option to reduce variability could be increasing replication, assuming equal bias toward one rot type versus another. This density bias is only a consideration if DAS indicates the historical presence of brown rot or if the L:D measure is used on all samples to infer nutritional mode.

### Legacy effects

Using material from the priority colonization trial, we demonstrated legacy effects but showed a discernable difference between rot types by including the Klason lignin measurement. As reported in Song et al. [44], birch substrate DAS nearly tripled over a 5-wk pre-colonization ‘priority’ period by a brown rot fungus (*G*. *trabeum*), reaching 58% at a mass loss of 11% (Table 2). In the 5 replicates we selected here having no detectable *G*. *trabeum* DNA after 8 weeks of competition with *I*. *lacteus*, DAS still reflected a brown rot legacy (63%) even though most mass had been lost (62% of total) while white rot was replacing brown rot. As a gauge of the influence of white rot, the L:D ratio was higher in *I*. *lacteus* dominated wood (L:D = 0.46) than when *G*. *trabeum* maintained dominance (L:D = 0.14). This increase of L:D as white rot became dominant was, however, not enough to push L:D over the 0.8 rot type threshold of wood-degrading fungi in pure culture, verifying the ‘drift’ mentioned in the Legacy Effects section, when there is a shift in rot type dominance.

**Table 2:** Legacy effects after brown rot (*Gloeophyllum trabeum*) 5-wk priority colonization on birch and subsequent 8-wk competition with white rot fungus (*Irpex lacteus*). Brown rot leaves a DAS imprint, even if *I*. *lacteus* comes to dominate, but lignin loss:density loss (L:D) ‘drifts.’

	Time Zero	Week 5	Week 13	
	No colonizer	*G*. *trabeum* priority	*G*. *trabeum* dominance	*I*. *lacteus* dominance
*G*. *trabeum* (%)^a^	None detected	100.0 (0.0)	90.6 (9.3)	0.2 (0.3)
Weight loss	NA	11.0 (4.5)	27.8 (3.0)	29.3 (4.9)
DAS (wt%)	20.3 (0.1)	57.9 (1.5)	68.2 (2.1)	62.7 (2.9)
Lignin (wt%)	32.9 (0.2)	NA	31.7 (0.6)	28.8 (0.4)
L:D^b^	NA	NA	0.14 (0.14)	0.46 (0.34)

^a^ *G*. *trabeum* (%) is from DNA copy counts, using isolate-specific primers as discussed in Song et al. [44].

^b^The L:D is used here and in the test as a field-applicable adaptation of Worrall et al. [4] lignin/weight loss (L/W) metric.

To overcome this mixed signature legacy in any scenario, L:D data can used as a stand-alone analysis rather than a simple complement for DAS. The L:D offers a scale of lignin selectivity ranging from slight (brown rot), to moderate (simultaneous white rot), to high (selective white rot), harboring information about the collective nutritional mode (Fig. 2). Unlike the rapid and irreversible brown rot effect on DAS, lignin removal is ongoing during decomposition. Here, when we observed *I*. *lacteus* replacing *G*. *trabeum* over 8 weeks, we saw a shift in L:D from 0.14 to 0.46 (Table 2). We know that *I*. *lacteus* causes simultaneous component loss [51] and we placed the L:D of *I*. *lacteus* around 0.9 in Song et al. [11] and 1.2 in this trial (Fig. 2a), with a difference likely due to decay stage (Fig. 2b). Combined, this would suggest a role for white rot of >50% in shaping the residues we recovered but with a relatively low lignin selectivity. If placed on a scale from 0 to 4.4, and knowing the DAS was high, this would indicate a role for brown rot and suggest relatively low lignin selectivity along the spectrum of nutritional modes.

### Field trial

**Fig 5:**
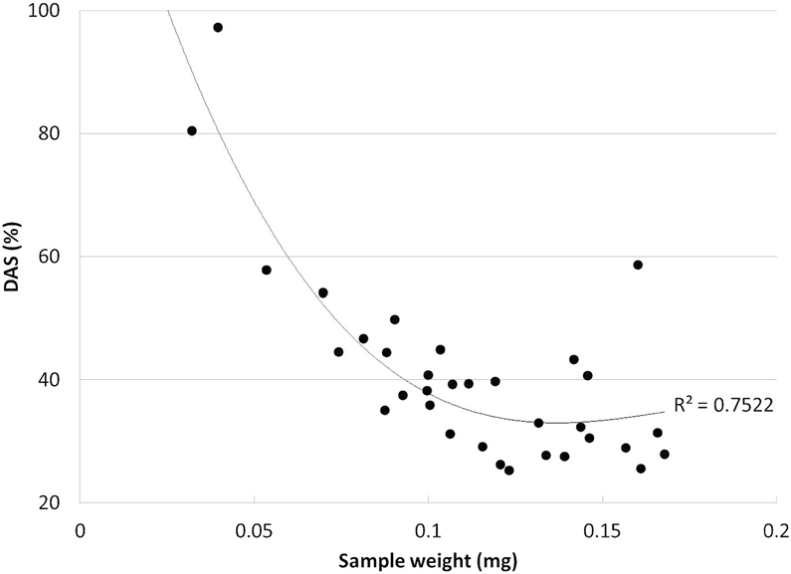
DAS sample size correlations. The DAS (wt%) values from wood collected with a 3/16ths Imperial drill bit from Radiata pine in New Zealand revealed obvious value inflation with smaller sample weights, a pattern shown here among all replicate samples including natural variability and some brown rot. This supports conservatively using more than 100 mg of material for DAS, as suggested by Shortle et al. 2012 [40].

In the New Zealand paired-plot study, we targeted decay class II/III logs for drill bit extractions and, likely as a consequence, found higher overall DAS levels and lower variability over a similar range (14.0–58.6) to those in a preliminary sampling attempt in Alaska (data not shown). The average DAS at the western plot was 36.9% (±9.8) and in the eastern plot pooled sample was 36.5%, suggesting a role for brown rot and indicating that the L:D method would have been useful to include. The coefficient of variability among technical replicates was 5.8%. Log-to-log variability was 26.6% in the western plot. The average CV within each log (n = 5 per log) was 23.1%, reflecting some spatial heterogeneity in fungal log colonization. Six samples from two decayed logs on the western site were not included in analyses, and the small amount of wood sawdust extractable in some cases with the drill bit showed a biasing effect on DAS, an important consideration in sample weight requirements that corroborates the 100–101 mg sample size used by Shortle et al. [40] (Fig. 5). If coupled with L:D, sample size requirements would be limited by Klason lignin needs, likely best with at least 1 g of field material (fresh wt).

Collectively, the results from this trial reinforces the decay class II/III target from the lab trials (given proper field identification when sampling logs) and it demonstrates how a preliminary survey can help match sampling approaches to replication needs in a given field setting. It also suggests that the drill extraction approach is viable, sampling or pooling sawdust within logs to meet sample size requirements for processing.

### Specific recommendations

We have demonstrated that both analyses (DAS and L:D) can provide complementary but distinct information about the dominant organisms decomposing wood. If the goal is to categorize the nutritional strategy of a wood-degrading fungus with high resolution, the L:D measurement will provide more information than the DAS. Conversely, if the goal is rapid assessment of rot type with high replication, the DAS will provide more data points than L:D in a set time frame. The DAS is an easier measurement than lignin on the benchtop, although not by much (~80 DAS versus 40 Klason samples per day). Both are low-cost, providing two community-relevant tools (shared here in Open Access) for researchers lacking funds, permits, facilities and/or equipment. In the case of field trials, DAS could provide a primary screen for wood rot type, followed by lignin analyses if there is an evident role for brown rot. If DAS is low and soft rot is not suspected (or implicated in a simplified pooled L:D test, etc.), white rot could be implicated outright. In any case, including and not necessarily limited to high DAS, a lignin analysis can place the predominant nutritional mode along a useful spectrum of L:D, allowing both incorporation of legacy effects and correlative statistics as a gradient (not binary) variable.

Specifically, we recommend extracting bark- and phloem-free wood [per 40] and delineating sap- from heartwood, if possible, within decay classes II and III ([46], Appendix 2 in [47]). Samples of known initial and final weights can be homogenized, and sample size requirement is >100 mg for DAS alone, but >1 g is best if incorporating L:D. An accurate density measure (via resistance drill) would be useful in deadwood. In either case, density is needed for L:D determination along with time zero lignin (preferable) or published lignin contents for sound wood per species if time zero material is lacking [52]. If decay is significant and DAS is less than 40%, white rot can generally be implicated, but we found that tree species and geographic variability warrant a preliminary assessment to establish statistical power. If DAS is higher than this threshold, a brown rot role is likely and measuring acid-insoluble lignin will provide L:D. One consideration for those working with unusually high-extractives woods (e.g. tropical heartwood), would be removing extractives in order to limit co-precipitation with lignin [53]. Collectively, the nutritional L:D spectrum would range from carbohydrate-selective (0.0) through the 0.8 brown/white rot dominance threshold and simultaneous white rot types toward lignin-selective white rot types (4.4 or higher). ‘Dominance’ would then harbor temporal information in a collective measure rather than dominance at one time point with unknown length of occupancy.

By following this approach, DAS and L:D can provide a more information-rich link between community structure and function than possible when measuring density loss, alone. As with any indirect measure of community or consequence, there must be caution in interpretation. On one hand, legacy effects could plausibly over-emphasize brown rot if using only DAS, while on the other hand faster brown rot rates (e.g. as a rule in standard method [54]) could bias for white rot dominance, instead. In a complex wood-inhabiting community, it is also possible that hydrolyzed carbohydrates of short chain length or lignin could be assimilated by non-degradative organisms in the community once released, although we did frequently measure high DAS in the field. Despite these and other caveats, these wood analyses add significant value as a residue measurement, providing more insight into decomposer biology and functional community history when coupled with density loss. With ever-expanding tools for biological and community analyses, alongside predictive models trying to integrate community effects with those of substrate and climate, this type of functional information can help bridge the gap between cause and consequence.
