# Correction: Signature Wood Modifications Reveal Decomposer Community History

**DOI:** 10.1371/journal.pone.0126877

**Published:** 2015-04-23

**Authors:** The PLOS ONE Staff

There are errors in the third column of Table 1 that were introduced during the typesetting process. Many of the rows in the third column incorrectly list *Antrodia vaillantii* instead of the many different *Genus species* that should be listed. Please view the correct Table 1 below.

The publisher apologizes for the errors.

**Table 1:** Fungal isolates used to assess strength of correlation between wood density loss on birch or pine and two dependent variables, 1) dilute alkali solubility (DAS) and 2) lignin:density loss (L:D).

Phylum^a^	Order	*Genus species* (original) revising author(s)	Isolate#^b^	Rot type^c^ (B clade)
Basidio	Polyporales	*Antrodia vaillantii* (DC.:Fr.) Ryv.	ATCC 11044	B (Antrod)
Basidio	Polyporales	*Fomitopsis cajanderi* (P. Karst.) Kotl. & Pouzar	A1-ATF	B (Antrod)
Basidio	Polyporales	*Fomitopsis pinicola* (Sw.:Fr.) P. Karst.	FP 105077R	B (Antrod)
Basidio	Polyporales	*Laetiporus sulphureus* (Bull.:Fr.) Murrill	TAB29	B (Antrod)
Basidio	Polyporales	*Piptoporus betulinus* (Bull.:Fr.) P. Karst.	A3-ATF	B (Antrod)
Basidio	Polyporales	*Postia placenta* (Fr.) Larsen & Lombard	MAD 698R	B (Antrod)
Basidio	Boletales	*Coniophora puteana* (Schum.:Fr.) P.Karst	ATCC 44393	B (Bolet)
Basidio	Boletales	*Leucogyrophana olivasceus* (Berk. & Curtis)	ATCC 22108	B (Bolet)
Basidio	Boletales	*Paxillus atrotomentosus* (Batsch:Fr.) Fr.	ATCC 64500	B (Bolet)
Basidio	Boletales	*Serpula himantiodes* (Fr.:Fr.) P. Karst.	ATCC 36335	B (Bolet)
Basidio	Boletales	*Serpula lacrymans* (Wulfen:Fr.) J. Schröt.	ATCC 82750	B (Bolet)
Basidio	Dacry-mycetales	*Dacryopinax* sp.	DJM 731	B (Dacry)
Basidio	Polyporales	*Gloeophyllum trabeum* (Pers.:Fr.) Murrill	ATCC 11539	B (Gloeo)
Basidio	Polyporales	*Wolfiporia cocos* (F.A. Wolf) Ryv. & Gil.	MD 104–5510	B (Wolfi)
Basidio	Polyporales	*Fomes fomentarius* L.:Fr.	BAM 001	W
Basidio	Polyporales	*Ganoderma australe* (Fr.:Fr.) Pat.	ATCC 90302	W
Basidio	Polyporales	*Irpex lacteus* (Fr.:Fr.) Fr.	ATCC 60993	W
Basidio	Polyporales	*Lenzites betulina* (L.:Fr.) Fr.	TAB356	W
Basidio	Hymeno-chaetales	*Phellinus igniarius* (L.:Fr.) Quel.	TAB386	W
Basidio	Agaricales	*Pleurotus ostreatus* (Jacq.:Fr.) P. Kumm.	ATCC 32237	W
Basidio	Polyporales	*Resinicium bicolor* (Alb. & Schwein.:Fr.) Parmasto	ATCC 44175	W
Basidio	Agaricales	*Schizophyllum commune* Fr.:Fr.	WFBDMN193	W
Basidio	Russulales	*Stereum hirsutum* (Willd.:Fr.) Gray	FP 91666	W
Basidio	Russulales	*Scytinostroma galactinum* (Fr.) Donk	ATCC 44175	W
Basidio	Polyporales	*Trametes versicolor* (L.:Fr.) Pilat	MAD 677R	W
Asco	Helotiales	*Cadophora luteo-olivacea* (Beyma) T.C. Harr. & McNew	Di90-5	S
Asco	Sordariales	*Chaetomium globosum* Kunze:Fr.	TAB91	S
Asco	Helotiales	*Phialocephala dimorphospora* Kendr.	Di28-3	S
Asco	Hypocreales	*Trichoderma viride* Pers.:Fr.	ATCC 32630	S

^a^Basidio—Basidiomycota; Asco—Ascomycota.

^b^Culture collections: ATCC—American Type Culture Collection, Manassas, VA, USA. FP and MAD—Center for Forest Mycology Research, U.S Forest Products Laboratory, Madison, WI, USA. All others—University of Minnesota Forest Pathology culture collection, Saint Paul, MN, USA.

^c^Wood rot types are from Gilbertson [9]. Brown rot clades are from Hibbett and Donoghue [31]. B-Brown rot; W-White rot; S-Soft rot. Clades are Antrodia, Boletales, Dacrymecetales, Gloeophyllum, and Wolfiporia.
